# Supplementary material for: Effects of wood fiber impulse-cyclone drying process on the UV-accelerated aging properties of wood-plastic composites
Source: PLoS One. 2022 Oct 27;17(10):e0266784. doi: 10.1371/journal.pone.0266784 (PMC9612545; doi:10.1371/journal.pone.0266784)
Supplement: S1 File — (DOCX) [file pone.0266784.s001.docx]

**Supporting Information**

Effects of Wood Fiber Impulse-Cyclone Drying Process on the UV-Accelerated Aging Properties of Wood-Plastic Composites

Qingde Li^1^, Feng Chen^2^*, Tonghui Sang^3&^

1. School of Industrial Design and Ceramic Art, Foshan University, Foshan, Guangdong, China
2. School of Art and Design, Taizhou University, Taizhou, Zhejiang, China
3. Faculty of creative technology and heritage, Universiti Malaysia Kelantan, Pengkalan Chepa, Kelantan, Malaysia

* Corresponding author

E-mail: chenfeng1984@tzc.edu.cn

^&^ These authors contributed equally to this work.

**S1 Table. Performance indicators of poplar fibers before and after drying treatment.**

Poplar residues were extracted via a wood fiber pulverizer (Model 60, Fuyang Energy Technology Co., Ltd., Xuzhou, Jiangsu, China). The poplar microfibers were then subjected to an impulse-cyclone dryer (Model MQG-50, Jianda Drying Equipment Co., Ltd., Changzhou, China) to prepare well-dispersed poplar fibers. The raw data for the performance indicators of poplar fibre before and after drying are shown in S1 Table.

**S1 Table .** Raw data of poplar fibers before and after drying treatment.

| **Performance indicator** | **Before drying (average)** | **Experimental data** | **After drying (average)** | **Experimental data** |
| --- | --- | --- | --- | --- |
| *Fiber morphology/mesh* | 60-80 |  | 60-80 |  |
| *Fiber length/mm* | 1.36(0.20) | 1.42 | 1.34(0.11) | 1.46 |
|  |  | 1.24 |  | 1.14 |
|  |  | 1.51 |  | 1.41 |
|  |  | 1.33 |  | 1.18 |
|  |  | 1.54 |  | 1.47 |
|  |  | 1.12 |  | 1.38 |
| *Fiber diameter/μm* | 223(31) | 222.83 | 211(21) | 211.91 |
|  |  | 223.55 |  | 211.50 |
|  |  | 222.48 |  | 211.91 |
|  |  | 223.78 |  | 211.28 |
|  |  | 223.44 |  | 210.13 |
|  |  | 221.92 |  | 209.27 |
| *Length–diameter ratio* | 6.1(1.87) | 5.59 | 6.3(0.67) | 7.15 |
|  |  | 6.51 |  | 5.35 |
|  |  | 5.29 |  | 5.78 |
|  |  | 5.93 |  | 6.11 |
|  |  | 6.84 |  | 5.6 |
|  |  | 6.44 |  | 7.81 |
| *Moisture content/%* | 10.6(0.19) | 11.58 | 2.3(0.09) | 2.20 |
|  |  | 11.18 |  | 2.43 |
|  |  | 11.18 |  | 2.09 |
|  |  | 9.85 |  | 2.42 |
|  |  | 11.28 |  | 2.47 |
|  |  | 8.53 |  | 2.19 |

Note: The results are means of measurements, and the numbers in brackets are the sample variances.

References

- Li, Q., Gao, X., Cheng, W., Han, G., & Han, J. (2017). Preparation and performance of high-density polyethylene-based wood–plastic composites reinforced with red pottery clay. Journal of Reinforced Plastics and Composites, 36(12), 853-863. <https://doi.org/10.1177/0731684417693698>
- Gao, X., Li, Q., Cheng, W., Han, G., & Xuan, L. (2018). Effects of moisture content, wood species, and form of raw materials on fiber morphology and mechanical properties of wood fiber‐HDPE composites. Polymer Composites, 39(9), 3236-3246. <https://doi.org/10.1002/pc.24336>

**S2 Table. Flexural strengths before and after UV-accelerated aging.**

The impact of UV-accelerated aging on the HDPE/wood fiber composites in outdoor environment was simulated according to the QUV test procedure. The specimens (76.2 mm × 76.2 mm × 3 mm) were placed into an accelerated weathering tester (QUV/SPRAY, Q-Panel Lab Products, Ohio, USA), and processed under an aging program set in accordance with ASTMG-154. A universal mechanical tester (WDW-20, Kexin Testing Instrument, Changchun, Jilin, China) was utilized to measure the mechanical properties of HDPE/wood fiber composites before and after accelerated aging. Through three-point bending test, the flexural strength of specimens (80 mm×13 mm×4 mm) was measured as per ASTM D 790 across a span of 80 mm (16 times the thickness of specimens) at a loading speed of 2.5 mm/min. The results of the experiments are shown in Table 4.

**S2 Table.** Raw data of flexural strengths.

| **Specimen No.** | **Flexural strength (MPa)** | | | | | | | |
| --- | --- | --- | --- | --- | --- | --- | --- | --- |
|  | **0h** | | **500h** | | **1000h** | | **1500h** | |
| *Specimen 1* | 53.16 | 52.95 | 52. 34 | 52.16 | 51. 96 | 52.26 | 50.46 | 51.11 |
|  |  | 53.73 |  | 52.17 |  | 52.01 |  | 50.96 |
|  |  | 52.3 |  | 52.15 |  | 52.12 |  | 49.55 |
|  |  | 53.65 |  | 51.74 |  | 52.48 |  | 49.53 |
|  |  | 53.19 |  | 51.45 |  | 51.74 |  | 51.3 |
|  |  | 53.14 |  | 54.37 |  | 51.15 |  | 50.31 |
| *Specimen 2* | 74.27 | 74.02 | 73.35 | 73.25 | 72.04 | 71.64 | 71.74 | 71.59 |
|  |  | 73.89 |  | 74.05 |  | 71.26 |  | 71.61 |
|  |  | 73.81 |  | 72.95 |  | 72.72 |  | 72.68 |
|  |  | 74.12 |  | 74.22 |  | 72.98 |  | 71.79 |
|  |  | 74.99 |  | 72.60 |  | 71.48 |  | 70.85 |
|  |  | 74.79 |  | 73.03 |  | 72.16 |  | 71.92 |
| *Specimen 3* | 75.82 | 76.66 | 74.68 | 75.15 | 73.71 | 72.81 | 72.73 | 72.86 |
|  |  | 75.93 |  | 75.03 |  | 73.85 |  | 73.60 |
|  |  | 75.34 |  | 74.20 |  | 74.48 |  | 72.69 |
|  |  | 75.02 |  | 74.51 |  | 73.24 |  | 73.72 |
|  |  | 76.75 |  | 75.64 |  | 74.21 |  | 73.27 |
|  |  | 75.22 |  | 73.55 |  | 73.67 |  | 70.24 |
| *Specimen 4* | 77.46 | 77.66 | 77.64 | 78.13 | 76.02 | 75.40 | 75.44 | 76.42 |
|  |  | 76.72 |  | 77.93 |  | 75.71 |  | 76.09 |
|  |  | 77.25 |  | 76.68 |  | 76.39 |  | 75.77 |
|  |  | 77.43 |  | 78.36 |  | 75.67 |  | 75.97 |
|  |  | 78.27 |  | 78.63 |  | 75.41 |  | 75.41 |
|  |  | 77.43 |  | 76.11 |  | 77.54 |  | 72.98 |
| *Specimen 5* | 71.14 | 70.30 | 70.04 | 70.75 | 69.46 | 68.97 | 68.36 | 68.92 |
|  |  | 71.95 |  | 69.70 |  | 69.93 |  | 67.75 |
|  |  | 72.09 |  | 70.77 |  | 68.99 |  | 68.26 |
|  |  | 71.04 |  | 69.44 |  | 69.14 |  | 68.66 |
|  |  | 71.65 |  | 69.46 |  | 68.67 |  | 67.99 |
|  |  | 69.81 |  | 70.12 |  | 71.06 |  | 68.58 |

| **Specimen No.** | **Flexural strength (MPa)** | | | | | |
| --- | --- | --- | --- | --- | --- | --- |
|  | **2000h** | | **2500h** | | **3000h** | |
| *Specimen 1* | 47.37 | 48.08 | 44.49 | 43.88 | 43.15 | 43.38 |
|  |  | 47.60 |  | 43.99 |  | 43.99 |
|  |  | 47.83 |  | 43.51 |  | 43.91 |
|  |  | 46.70 |  | 44.75 |  | 42.31 |
|  |  | 48.16 |  | 44.29 |  | 44.02 |
|  |  | 45.85 |  | 46.52 |  | 41.29 |
| *Specimen 2* | 68.12 | 68.94 | 65.67 | 65.28 | 62.08 | 62.41 |
|  |  | 67.66 |  | 66.17 |  | 61.66 |
|  |  | 68.20 |  | 65.47 |  | 62.70 |
|  |  | 67.20 |  | 66.18 |  | 62.36 |
|  |  | 67.33 |  | 65.50 |  | 62.55 |
|  |  | 69.39 |  | 65.42 |  | 60.80 |
| *Specimen 3* | 69.42 | 70.39 | 66.38 | 66.37 | 63.45 | 63.99 |
|  |  | 69.61 |  | 65.67 |  | 63.35 |
|  |  | 69.29 |  | 65.40 |  | 63.65 |
|  |  | 70.37 |  | 65.72 |  | 62.65 |
|  |  | 69.01 |  | 66.70 |  | 64.07 |
|  |  | 67.85 |  | 68.42 |  | 62.99 |
| *Specimen 4* | 72.74 | 72.28 | 68.03 | 68.35 | 65.40 | 66.35 |
|  |  | 72.02 |  | 68.73 |  | 64.41 |
|  |  | 73.44 |  | 67.58 |  | 65.80 |
|  |  | 73.33 |  | 68.52 |  | 64.46 |
|  |  | 72.52 |  | 68.37 |  | 64.98 |
|  |  | 72.85 |  | 66.63 |  | 66.40 |
| *Specimen 5* | 65.06 | 65.52 | 62.84 | 61.86 | 59.63 | 60.28 |
|  |  | 65.62 |  | 62.3 |  | 60.00 |
|  |  | 64.42 |  | 63.52 |  | 60.09 |
|  |  | 65.83 |  | 63.00 |  | 59.67 |
|  |  | 64.31 |  | 62.57 |  | 58.67 |
|  |  | 64.66 |  | 63.79 |  | 59.07 |

References

- Li, Q., Gao, X., Cheng, W., Han, G. (2017). Effect of Modified Red Pottery Clay on the Moisture Absorption Behavior and Weather ability of Polyethylene-Based Wood-Plastic Composites. Materials, 10(111), 2-17. Doi:10.3390/ma10020111.

**S3 Table. Flexural modulus before and after UV-accelerated aging.**

Table 5 illustrates the variation patterns of flexural moduli for HDPE/wood fiber composites. The wood fibers in specimens 1-5 were treated by impulse-cyclone drying and modified with A187 separately under the following conditions: 180°C, 180°C + A187, 200°C + A187, 220°C + A187, and 240°C + A187.

**S3 Table.** Raw data of flexural modulus.

| **Specimen No.** | **Flexural modulus (MPa)** | | | | | | | |
| --- | --- | --- | --- | --- | --- | --- | --- | --- |
|  | **0h** | | **500h** | | **1000h** | | **1500h** | |
| *Specimen 1* | 2427.01 | 2427.11 | 2378.63 | 2378.23 | 2299.66 | 2298.87 | 2219.79 | 2219.00 |
|  |  | 2426.40 |  | 2378.91 |  | 2300.34 |  | 2220.32 |
|  |  | 2426.89 |  | 2378.95 |  | 2299.80 |  | 2219.35 |
|  |  | 2426.74 |  | 2378.32 |  | 2300.14 |  | 2219.62 |
|  |  | 2426.27 |  | 2377.82 |  | 2299.26 |  | 2219.26 |
|  |  | 2428.65 |  | 2379.55 |  | 2299.55 |  | 2221.19 |
| *Specimen 2* | 2489.34 | 2489.77 | 2483.45 | 2484.08 | 2385.34 | 2386.06 | 2286.99 | 2286.57 |
|  |  | 2488.68 |  | 2483.43 |  | 2385.43 |  | 2286.27 |
|  |  | 2488.67 |  | 2482.73 |  | 2384.77 |  | 2286.34 |
|  |  | 2489.56 |  | 2483.98 |  | 2384.50 |  | 2286.42 |
|  |  | 2489.36 |  | 2483.36 |  | 2386.19 |  | 2287.35 |
|  |  | 2490.00 |  | 2483.12 |  | 2385.09 |  | 2288.99 |
| *Specimen 3* | 2525.37 | 2524.89 | 2535.98 | 2535.15 | 2442.92 | 2443.49 | 2334.22 | 2334.48 |
|  |  | 2525.06 |  | 2535.03 |  | 2442.77 |  | 2335.06 |
|  |  | 2526.14 |  | 2535.54 |  | 2443.75 |  | 2334.91 |
|  |  | 2526.14 |  | 2536.86 |  | 2443.47 |  | 2333.77 |
|  |  | 2525.23 |  | 2536.26 |  | 2442.73 |  | 2335.19 |
|  |  | 2524.76 |  | 2537.04 |  | 2441.31 |  | 2331.91 |
| *Specimen 4* | 2543.11 | 2542.71 | 2548.38 | 2547.68 | 2444.32 | 2443.60 | 2334.43 | 2334.95 |
|  |  | 2543.18 |  | 2548.92 |  | 2444.10 |  | 2335.36 |
|  |  | 2542.13 |  | 2548.98 |  | 2443.34 |  | 2333.55 |
|  |  | 2543.39 |  | 2547.66 |  | 2443.63 |  | 2333.93 |
|  |  | 2542.85 |  | 2549.18 |  | 2444.00 |  | 2333.97 |
|  |  | 2544.40 |  | 2547.86 |  | 2447.25 |  | 2334.82 |
| *Specimen 5* | 2469.84 | 2470.74 | 2402.32 | 2403.29 | 2358.93 | 2359.60 | 2274.33 | 2273.96 |
|  |  | 2469.23 |  | 2401.67 |  | 2359.38 |  | 2274.53 |
|  |  | 2470.11 |  | 2401.86 |  | 2359.17 |  | 2275.23 |
|  |  | 2470.74 |  | 2401.79 |  | 2358.49 |  | 2273.54 |
|  |  | 2470.46 |  | 2402.86 |  | 2359.73 |  | 2275.13 |
|  |  | 2467.76 |  | 2402.45 |  | 2357.21 |  | 2273.59 |

| **Specimen No.** | **Flexural modulus (MPa)** | | | | | |
| --- | --- | --- | --- | --- | --- | --- |
|  | **2000h** | | **2500h** | | **3000h** | |
| *Specimen 1* | 2087.37 | 2086.89 | 1954.49 | 1954.90 | 1822.15 | 1821.28 |
|  |  | 2086.82 |  | 1954.65 |  | 1821.77 |
|  |  | 2086.72 |  | 1953.70 |  | 1821.68 |
|  |  | 2086.77 |  | 1954.14 |  | 1822.73 |
|  |  | 2086.91 |  | 1954.31 |  | 1821.35 |
|  |  | 2090.11 |  | 1955.24 |  | 1824.09 |
| *Specimen 2* | 2160.12 | 2161.10 | 2033.67 | 2033.53 | 1947.08 | 1947.56 |
|  |  | 2159.62 |  | 2034.11 |  | 1947.10 |
|  |  | 2159.37 |  | 2034.66 |  | 1946.36 |
|  |  | 2160.68 |  | 2033.41 |  | 1947.19 |
|  |  | 2160.64 |  | 2032.99 |  | 1946.95 |
|  |  | 2159.31 |  | 2033.32 |  | 1947.32 |
| *Specimen 3* | 2217.61 | 2217.41 | 2101.75 | 2101.35 | 2004.17 | 2003.28 |
|  |  | 2217.76 |  | 2101.27 |  | 2004.71 |
|  |  | 2217.60 |  | 2102.75 |  | 2003.98 |
|  |  | 2218.57 |  | 2101.09 |  | 2003.82 |
|  |  | 2216.63 |  | 2101.01 |  | 2004.22 |
|  |  | 2217.69 |  | 2103.03 |  | 2005.01 |
| *Specimen 4* | 2290.89 | 2291.73 | 2146.20 | 2146.88 | 2082.08 | 2082.93 |
|  |  | 2291.86 |  | 2147.09 |  | 2083.07 |
|  |  | 2290.72 |  | 2145.59 |  | 2082.17 |
|  |  | 2290.03 |  | 2145.25 |  | 2082.77 |
|  |  | 2290.07 |  | 2146.29 |  | 2081.45 |
|  |  | 2290.93 |  | 2146.10 |  | 2080.09 |
| *Specimen 5* | 2121.16 | 2121.63 | 2018.97 | 2018.19 | 1914.20 | 1914.92 |
|  |  | 2120.27 |  | 2018.70 |  | 1914.25 |
|  |  | 2121.68 |  | 2018.91 |  | 1915.1 |
|  |  | 2120.22 |  | 2019.29 |  | 1914.44 |
|  |  | 2120.46 |  | 2018.77 |  | 1913.31 |
|  |  | 2122.70 |  | 2019.96 |  | 1913.18 |

References

- Li, Q., Gao, X., Cheng, W., Han, G. (2017). Effect of Modified Red Pottery Clay on the Moisture Absorption Behavior and Weather ability of Polyethylene-Based Wood-Plastic Composites. Materials, 10(111), 2-17. Doi:10.3390/ma10020111.
- Gao, X.; Li, Q.; Cheng, W.; Han, G.; Xuan, L. Optimization of High Temperature and Pressurized Steam Modified Wood Fibers for High-Density Polyethylene Matrix Composites Using the Orthogonal Design Method. *Materials* 2016, 9, 847. <https://doi.org/10.3390/ma9100847>

**S4 Table. Impact strengths before and after UV-accelerated aging.**

A universal mechanical tester (WDW-20, Kexin Testing Instrument, Changchun, Jilin, China) was utilized to measure the mechanical properties of HDPE/wood fiber composites before and after accelerated aging. Impact strength was measured through the simply supported beam pendulum impact test in accordance with ASTM D256. The specimen dimensions were 80 mm×10 mm×4 mm, while the pendulum impact tester (XJC-25, Chengde Precision Testing Machine Co., Ltd.) was set to a span of 60 mm, a pendulum energy of 2 J, and an impact velocity of 2.9 m/s. The results of the experiments are shown in Table 6.

**S4 Table .** Raw data of impact strength.

| **Specimen No.** | **Impact strength ( KJ·m^-2^)** | | | | | | | |
| --- | --- | --- | --- | --- | --- | --- | --- | --- |
|  | **0h** | | **500h** | | **1000h** | | **1500h** | |
| *Specimen 1* | 11.29 | 11.52 | 11.04 | 11.56 | 10.97 | 2298.87 | 10.73 | 10.27 |
|  |  | 11.53 |  | 11.82 |  | 2300.34 |  | 11.02 |
|  |  | 12.06 |  | 11.43 |  | 2299.80 |  | 9.85 |
|  |  | 11.08 |  | 11.23 |  | 2300.14 |  | 10.64 |
|  |  | 10.53 |  | 10.20 |  | 2299.26 |  | 11.10 |
|  |  | 11.02 |  | 9.58 |  | 2299.55 |  | 11.50 |
| *Specimen 2* | 13.04 | 13.46 | 13.12 | 12.72 | 13.02 | 12.35 | 12.50 | 12.35 |
|  |  | 12.21 |  | 12.89 |  | 13.53 |  | 12.91 |
|  |  | 12.91 |  | 12.21 |  | 13.40 |  | 12.39 |
|  |  | 13.00 |  | 13.31 |  | 13.19 |  | 12.80 |
|  |  | 13.38 |  | 13.30 |  | 12.48 |  | 12.30 |
|  |  | 13.28 |  | 14.29 |  | 13.17 |  | 12.25 |
| *Specimen 3* | 13.91 | 13.26 | 13.47 | 13.27 | 13.12 | 14.02 | 13.33 | 12.76 |
|  |  | 14.70 |  | 13.13 |  | 14.03 |  | 13.18 |
|  |  | 13.22 |  | 14.38 |  | 13.87 |  | 12.93 |
|  |  | 13.79 |  | 13.96 |  | 13.15 |  | 14.10 |
|  |  | 14.30 |  | 13.31 |  | 12.24 |  | 12.93 |
|  |  | 14.19 |  | 12.77 |  | 11.41 |  | 14.08 |
| *Specimen 4* | 14.30 | 13.83 | 14.40 | 14.28 | 14.32 | 13.76 | 13.72 | 12.79 |
|  |  | 14.23 |  | 14.72 |  | 15.22 |  | 12.85 |
|  |  | 14.36 |  | 14.11 |  | 14.64 |  | 14.03 |
|  |  | 14.08 |  | 14.71 |  | 15.03 |  | 13.98 |
|  |  | 13.57 |  | 14.25 |  | 14.50 |  | 13.67 |
|  |  | 15.73 |  | 14.33 |  | 12.77 |  | 15.00 |
| *Specimen 5* | 11.61 | 11.42 | 11.48 | 11.23 | 11.28 | 12.27 | 11.09 | 10.75 |
|  |  | 11.52 |  | 11.38 |  | 11.72 |  | 11.65 |
|  |  | 10.88 |  | 10.51 |  | 11.60 |  | 10.46 |
|  |  | 12.30 |  | 10.52 |  | 12.02 |  | 11.38 |
|  |  | 12.55 |  | 10.68 |  | 10.79 |  | 11.78 |
|  |  | 10.99 |  | 14.56 |  | 9.28 |  | 10.52 |

| **Specimen No.** | **Impact strength ( KJ·m^-2^)** | | | | | |
| --- | --- | --- | --- | --- | --- | --- |
|  | **2000h** | | **2500h** | | **3000h** | |
| *Specimen 1* | 10.47 | 10.88 | 10.21 | 10.17 | 9.95 | 9.09 |
|  |  | 9.98 |  | 10.80 |  | 10.91 |
|  |  | 9.81 |  | 9.56 |  | 10.19 |
|  |  | 10.37 |  | 9.53 |  | 10.65 |
|  |  | 11.08 |  | 11.11 |  | 9.56 |
|  |  | 10.70 |  | 10.09 |  | 9.30 |
| *Specimen 2* | 12.05 | 12.33 | 11.20 | 10.79 | 11.65 | 11.98 |
|  |  | 12.06 |  | 10.95 |  | 12.32 |
|  |  | 11.23 |  | 11.74 |  | 12.37 |
|  |  | 11.43 |  | 10.67 |  | 12.09 |
|  |  | 11.16 |  | 11.59 |  | 10.68 |
|  |  | 14.09 |  | 11.46 |  | 10.46 |
| *Specimen 3* | 13.04 | 12.81 | 12.77 | 13.16 | 12.47 | 12.32 |
|  |  | 12.79 |  | 13.68 |  | 12.12 |
|  |  | 12.07 |  | 13.10 |  | 12.14 |
|  |  | 12.77 |  | 12.89 |  | 12.17 |
|  |  | 12.50 |  | 12.17 |  | 13.41 |
|  |  | 15.30 |  | 11.62 |  | 12.66 |
| *Specimen 4* | 13.44 | 14.34 | 13.13 | 12.66 | 12.85 | 12.77 |
|  |  | 13.77 |  | 13.99 |  | 12.54 |
|  |  | 13.84 |  | 12.80 |  | 12.72 |
|  |  | 13.33 |  | 12.48 |  | 12.51 |
|  |  | 13.29 |  | 13.26 |  | 12.20 |
|  |  | 12.07 |  | 13.59 |  | 14.36 |
| *Specimen 5* | 10.87 | 11.36 | 10.59 | 10.45 | 10.25 | 10.10 |
|  |  | 11.32 |  | 10.38 |  | 10.22 |
|  |  | 10.76 |  | 10.62 |  | 10.33 |
|  |  | 11.41 |  | 10.26 |  | 9.99 |
|  |  | 11.29 |  | 11.49 |  | 9.73 |
|  |  | 9.08 |  | 10.34 |  | 11.13 |

References

- F. Chen, G. Han, Q. Li, X. Gao, W. Cheng, High-Temperature Hot Air/Silane Coupling Modification of Wood Fiber and Its Effect on Properties of Wood Fiber/HDPE Composites, Journal of Materials, 10 (2017), 286-303. doi:10.3390/ma10030286

**S5 Table. Surface chromaticity changes after UV-accelerated aging.**

A spectrophotometer (CM-2300d, Konica Minolta, Japan) was utilized to measure the surface chromaticity values of HDPE/wood fiber composite specimens. The *L***a***b** color system developed by the International Commission of Illumination CIE (1976) was used for color notation, where the data comprised luminosity values (*L**) and two chromaticity coordinates (*a** and *b**). The changes in color could be expressed by formulas (1) and (2):

*△E**=（*△L**^2^+*△a**^2^+*△b**^2^） ^1/2^ （1）

*△L**= *L**- *L_0_** （2）

*△a**= *a**- *a_0_** （3）

*△b**= *b**- *b_0_** （4）

**S5 Table.** Raw data of surface chromaticity changes after UV-accelerated aging.

| **Specimen No.** | **Degree of luminosity change (*△L**)** | | | | | | |
| --- | --- | --- | --- | --- | --- | --- | --- |
|  | **0h** | **500h** | **1000h** | **1500h** | **2000h** | **2500h** | **3000h** |
| *Specimen 1* | 0 | 6.504 | 8.097 | 20.073 | 23.047 | 24.021 | 24.995 |
| *Specimen 2* | 0 | 6.912 | 9.302 | 16.250 | 21.705 | 22.120 | 22.565 |
| *Specimen 3* | 0 | 6.647 | 7.812 | 15.333 | 19.604 | 21.277 | 21.747 |
| *Specimen 4* | 0 | 6.440 | 7.432 | 14.172 | 18.544 | 20.013 | 20.636 |
| *Specimen 5* | 0 | 6.748 | 8.812 | 18.609 | 22.587 | 23.059 | 23.825 |

| **Specimen No.** | **Degree of discoloration (*△E**)** | | | | | | |
| --- | --- | --- | --- | --- | --- | --- | --- |
|  | **0h** | **500h** | **1000h** | **1500h** | **2000h** | **2500h** | **3000h** |
| *Specimen 1* | 0 | 5.637 | 7.802 | 21.109 | 24.404 | 25.313 | 25.995 |
| *Specimen 2* | 0 | 6.967 | 9.228 | 17.033 | 22.015 | 23.120 | 23.165 |
| *Specimen 3* | 0 | 5.806 | 8.512 | 15.750 | 21.304 | 22.737 | 22.147 |
| *Specimen 4* | 0 | 5.432 | 7.097 | 14.073 | 20.467 | 21.021 | 21.845 |
| *Specimen 5* | 0 | 6.383 | 9.032 | 19.572 | 23.087 | 24.059 | 24.025 |

References

- Xuan, L., Han, G., Wang, D., Cheng, W., Gao, X., Chen, F., & Li, Q. (2017). Effect of surface-modified TiO2 nanoparticles on the anti-ultraviolet aging performance of foamed wheat straw fiber/polypropylene composites. Materials, 10(5), 456. <https://doi.org/10.3390/ma10050456>
- Li, Q., Gao, X., Cheng, W., Han, G. (2017). Effect of Modified Red Pottery Clay on the Moisture Absorption Behavior and Weather ability of Polyethylene-Based Wood-Plastic Composites. Materials, 10(111), 2-17. Doi:10.3390/ma10020111.

**S1 Fig. Impulse-cyclone dryer.**

As shown in Fig 1, the impulse-cyclone drying treatment featured short duration, high strength, high efficiency, simple process and environmental friendliness. At the beginning of the drying test, warm air was injected into the (1) electric heater to preheat the drying system. After the system was filled with hot air and inlet temperature was constant, the (9) screw feeder was used to begin pressing the wood fibers into the (2) impulse dryer. Under the accelerating and decelerating motion of wood fibers in the impulse dryer, free water in wood cell cavity is quickly vaporized within the impulse dryer. The fibers were allowed to flow into the (3) cyclone dryer to remove the water bound to the surface of the pulp, which was difficult to dry. The larger fibers were collected by the (8) air-off layout collector and the finer particles entered the (5) dust collector with the exhaust gas. In order to verify the temperature change and airflow velocity change in ICAD system during the simulated drying process, temperature measuring thermocouples and velocity sensors were placed at the inlet, straight tube, impulse tube, middle and gas outlet of the drying system, (shown by A, B, C, D and E in S1 Fig).


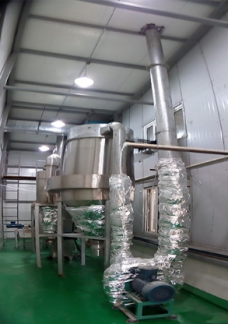

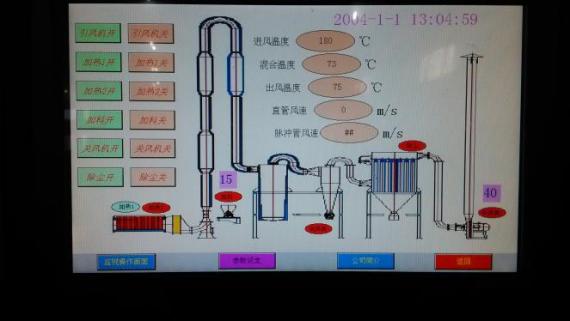

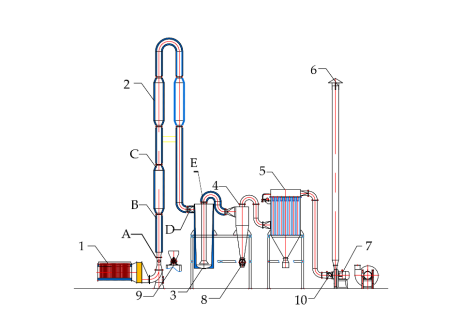


**S1 Fig . Impulse-cyclone dryer and Operating platform**

(1. heater; 2. Impulse bag filter; 3. Cyclone separator; 4. Cyclone dryer; 5. Impulse dryer; 6. Helical feeder; 7. Air inlet pipe; 8. Air filter; 9. Air regulating valve; 10. Fan; 11. Feed port; 12. Air inlet port; 13. Exhaust port; 14. Closed air discharger; 15. Discharge port; 16. Branch pipe; 17. Exhaust pipe; 18. PCL controller; 19. Heater)

References

- Feng C., Li Q., Xun G., Han G., Cheng W. Impulse-cyclone Drying Treatment of Poplar Wood Fibers and its Effect on Composite Material's Properties. Bioresources. 2017;12(2): 3948-3964. DOI: 10.15376/biores.12.2.3948-3964

**S2 Fig. The surface microstructure of UV-accelerated aged composites**

During the experiment, the samples were prepared into smaller blocks with cutter, placed onto the sample holder while trying to keep the upward surfaces smooth. After firmly fixing both sides of samples with carbon conductive adhesive, the samples were sputtered with gold layer in a vacuum coater. The experimental process was implemented in accordance with ASTM E1588 (2017), and the sample morphological characteristics were observed using QUANTA 200 SEM system under an accelerating voltage of 30 kV. The results of this experimental test data were all done independently by the authors and the test procedure is shown in Figure 7.


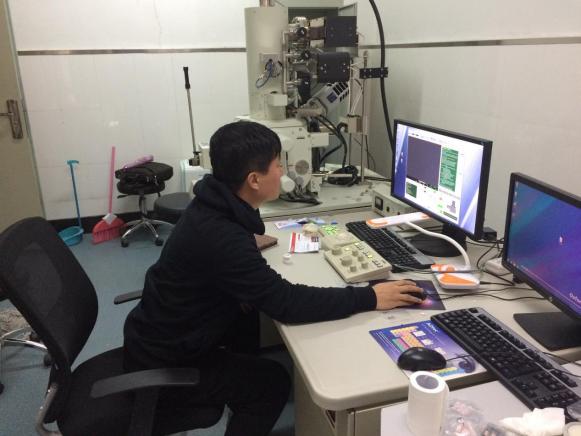


**S2 Fig.** Surface microstructures after UV-accelerated aging（Mag-2000X）. The author was operating the experimental apparatus.

References

- Li, Q., Liang, Y., Chen, F., and Sang, T. (2020). "Preparation and performance of modified montmorillonite-reinforced wood-based foamed composites," BioRes. 15(2), 3566-3584. DOI：10.15376/biores.15.2.3566-3584
